# Uncertainty quantification of multi-scale resilience in networked systems with nonlinear dynamics using arbitrary polynomial chaos

**DOI:** 10.1038/s41598-022-27025-w

**Published:** 2023-01-10

**Authors:** Mengbang Zou, Luca Zanotti Fragonara, Song Qiu, Weisi Guo

**Affiliations:** 1 School of Aerospace Transport and Manufacturing, Cranfield University, Cranfield, MK43 0AL UK; 2 Alan Turing Institute, London, NW1 2DB UK; 3 Present Address: School of Physical Science and Technology, Southwest Jiaotong University, Chengdu, 610032 China

**Keywords:** Complex networks, Nonlinear phenomena

## Abstract

Complex systems derive sophisticated behavioral dynamics by connecting individual component dynamics via a complex network. The resilience of complex systems is a critical ability to regain desirable behavior after perturbations. In the past years, our understanding of large-scale networked resilience is largely confined to proprietary agent-based simulations or topological analysis of graphs. However, we know the dynamics and topology both matter and the impact of model uncertainty of the system remains unsolved, especially on individual nodes. In order to quantify the effect of uncertainty on resilience across the network resolutions (from macro-scale network statistics to individual node dynamics), we employ an arbitrary polynomial chaos (aPC) expansion method to identify the probability of a node in losing its resilience and how the different model parameters contribute to this risk on a single node. We test this using both a generic networked bi-stable system and also established ecological and work force commuter network dynamics to demonstrate applicability. This framework will aid practitioners to both understand macro-scale behavior and make micro-scale interventions.

## Introduction

In a connected world, local component dynamics contribute collectively to system-of-system behaviour. This can be observed in engineered critical infrastructure (CI) systems^1^, ecosystems^2^, biological systems^3^ etc. Components of the complex system affect each other through interactions among them and all together result in a more sophisticated multi-scale network wide dynamics. One important part of the system’s behaviors is the resilience which is defined as the ability of a system to maintain its original functionality when perturbations happen^2^. This ability is of great importance in reducing risks and mitigating damage^4,5^. Research on resilience of networks has attracted a lot of attention in recent years in a wide range of fields from nature to man-made networks, including blackouts in power systems^6^, failure of water supply system^7^ to loss of biodiversity in ecology^8^. Whilst the past research on resilience gives us insight into how a few interacting components (small networks) work^5^, the loss of resilience in large-scale networked systems (e.g. $10^5$ nodes) is difficult to predict and analyze analytically. Current industrial standards use high fidelity agent-based simulations, but these lack a generalizable understanding on how and why network topology and dynamics combine to contribute to resilience and how uncertainty affect the resilience.

### Review on multi-scale resilience of complex systems

Many performance-based methods have been proposed to quantify the macro resilience of systems in different domains with different metrics^9,10^. But the loss of resilience of complex system with large number of components in complex natural and man-made system is still difficult to predict. The limitation is that the most analytical framework of resilience is designed to treat low-dimensional models with a few interacting components^2^. In a high-dimensional system with large number of interacting components, the network topology of the system plays an important role in dynamic behaviors. Traditional methods pay little attention to the importance of the network topology of the system. Therefore, these methods are difficult to analyze the resilience of the networked system when perturbations happen to the network topology. To solve this problem, a network-based theoretical framework was developed to analyze the resilience of the multi-dimensional system consisting of a lot of interacting components. They reduced the multi-dimensional dynamics equations of the system to an effective one-dimensional equation to analyze the macro-scale (network-level) resilience according to the network topology of the system^2^. Whilst this has given us insight into the coupling relationship between topology and dynamics, the effect of individual nodes on system’s resilience is still not clear. To break this limit, a new centrality index, resilience centrality, is derived in^11^ to quantify the ability of nodes to affect the resilience of the system. However, these methods are still unable to make the prediction of micro-scale (node-level) dynamics. Node level is important to make critical interventions to specific components while preserving our multi-scale (macro and micro scale) understanding of general system behaviors. To precisely identify the node-level resilience function, a sequential heterogeneous estimation approach is proposed recently^12^. In^12^, it is assumed that the model of the system is certain and all of the information is known. Yet, in a real system, uncertainty may exist. How does the uncertainty affect the resilience of system from macro-scale and micro-scale is not clear.

### Review on uncertainty in multi-scale resilience of complex systems

Model uncertainty may stem directly from the incomplete information of the system or measurement noise of the initial data as well as from parameters of models whose values are not known exactly^13,14^. In order to know the effect of uncertain parameters on the network-level resilience, previous work introduced a polynomial chaos method^15^ to understand network-level resilience loss with uncertainty. Uncertainty not only affects the macro-level resilience but also can affect individual nodes’, especially uncertainty may cause different effects on different nodes in a network. This can paint a different picture to that of the overall macro-scale network behavior found in previous work^15^. That is to say, a macro-scale resilient network may hide non-resilient behavior at the micro-scale, which if not addressed in time can cause long term issues. Methods to analyze the resilience of system are summarized in Table 1. Comparing with previous methods, we analyze both the macro and micro scale resilience of high dimensional system consisting of interacting components as well as the effects of uncertainty on multi-scale resilience.

Since uncertainty is widespread in practical problems in the real world and has an effect on systems’ performance, how to quantify these uncertain factors is the main purpose of research on Uncertainty Quantification (UQ). UQ methods mainly include: Monte Carlo methods^16^, perturbation methods^17^, moment equation methods^18^, polynomial approximation methods^19^. Due to the high accuracy and computational efficiency comparing with traditional UQ methods like Monte Carlo methods, polynomial chaos expansion (PCE) methods have been widely used in dynamic systems^20^. For example, a PCE method was used to estimate the dynamic response bounds of nonlinear system with interval uncertainty^21^ and to analyze the effect of uncertainty in parameters on the received signal concentration in molecular signals^22^. The PCE was initially proposed to analyze stochastic processes based on Hermite polynomials, which were only suitable for random variables (r.v.) following Gaussian distribution^19^. However, uncertainty does not always obey the Gaussian distribution. While a normal score transformation could be used to solve this problem^23^, it will lead to slow convergence^24^. To solve this problem, the generalized polynomial chaos (gPC) has been developed^24,25^. The gPC extends PCE toward a broader range of applications which could be used to encompass the more general Gamma distribution, Beta distribution, and many other flexible distribution functions. This is further advanced to consider stochastic processes represented by r.v. of any probability distributions^26^.

The methods mentioned above need to know the detailed information of the involved probability density functions (PDF). However, information about distribution is usually difficult to know or incomplete in practical applications. In different models or circumstances, statistical information of parameters may exist in many different forms. They could be discrete, continuous, or discretized continuous, even exist analytically in the probability density distribution or numerically as a histogram. There are two main reasons that limit the widespread use of the above methods. The first reason is that there exist strict restrictions in most cases when they are used. The second is that the information of problems to be solved is not always complete and perfect^27^. When quantifying the uncertainty on the network-level resilience, mean field estimation and Central Limit Theorem (CLT) can be employed to make the system’s equilibrium depend on a r.v. following Gaussian distribution^15^. So, the PCE methods can be directly used. However, when estimating the node-level resilience, CLT is not suitable to be used. In this situation, if parameters of the system do not obey distributions as mentioned above, the gPC is no longer effective. To quantify the uncertainty following arbitrary probability distributions, arbitrary polynomial chaos (aPC) method has been proposed^27^. Therefore, this method can be employed to quantify uncertainty of individual node’s resilience.

**Table 1:** Methods to analyze resilience of system.

Method	Dimension of system	Interacting of components	Network-level resilience	Node-level resilience	Uncertainty of system	Limitations	References
Performance- based methods with metrics	Low; captured by a one-dimensional nonlinear dynamic equation or time- series data analysis without modelling	No	Yes	No	No	Not suitable to high dimensional system with interacting components; not consider network topology	^9,10^^28–30^
Network-based method	High; consider ODE dynamics of each component of system	Yes; interaction is characterized by ODE	Yes	No	No	Cannot estimate resilience of individual nodes; not consider effects of uncertainty	^2,11^^31^
Sequential estimation method	High; consider ODE dynamics of each component of system	Yes; interaction is characterized by ODE	No	Yes	No	Not consider effects of uncertainty on node- level and network-level resilience	^12^
Specific network- based method with PCE	High; consider ODE dynamics of each component of system	Yes; interaction is characterized by ODE	Yes	No	Yes	Not consider effects of uncertainty on node-level resilience; need to know the distribution of uncertain parameters	^15^
Specific sequential estimation method with aPC	High; consider ODE dynamics of each component of system	Yes; interaction is characterized by ODE	Yes	Yes	Yes	Not suitable for networked system with PDE dynamics	This paper

### Novelty and contribution

We have listed different methods to analyze resilience of system in Table 1 and compared them with this paper from different aspects. Our previous work has explored how to estimate the resilience of networked system with interacting components. In^12^, a sequential estimation method is proposed to estimate the node-level resilience. It is assumed that all parameters of the system model are known and determined. However, uncertainty, which may stem from incomplete information of the system or measurement noise of the initial data as well as parameters of model not known exactly, is widespread in practical problems in real world and affects performance of the system. To quantify the effects of uncertainty on the network-level resilience of the system, a specific network-based method with PCE is employed in^15^. However, it was not clear before our new paper:

(1) how uncertainty of the system model affects the performance of each node. Similar network-level dynamic behavior may hide different individual node behavior. Therefore, research on network-level and node-level behavior are both important for us to comprehensively understand the networked system;

(2) how global network topology and local network topology affect the resilience of individual node with the existing of system uncertainty.

The novelty of this paper comparing with previous work is that a specific sequential estimation method with multi-dimensional aPC is employed to quantify uncertain factors, which addresses the lack of uncertainty quantification in the multi-scale resilience (network-level and node-level) of complex networks with nonlinear dynamics. The use of aPC enable parameter uncertainty that follows arbitrary distributions, which makes our method a wide range of application scenarios.

Another contribution is that we build the relationship between network topology and node-level resilience with uncertainty. We find that when the average degree across network (global topology) is greater than a high critical value (threshold value of critical weight), all of the nodes are resilient, and the uncertainty of the system will not affect the resilience. The critical value is useful in practical applications to help predict the resilience of the system. For example, in a work force commuter network, the weight of a city is decided by the number of services for commuting to this city. If the average number of services by rail transport links of the system is greater than the threshold value of critical weight, then the working population of all cities is resilient. Otherwise, the probability to be resilient of each node is decided by both global and local topology (average degree across network and in-degree of each node). The establishment of this relationship help us better understand the effects of network topology on macro and micro behavior of the networked system and also enable us to predict the impact of perturbations on macro and micro resilience of the system according to the global and local network topology.

**Figure 1:**
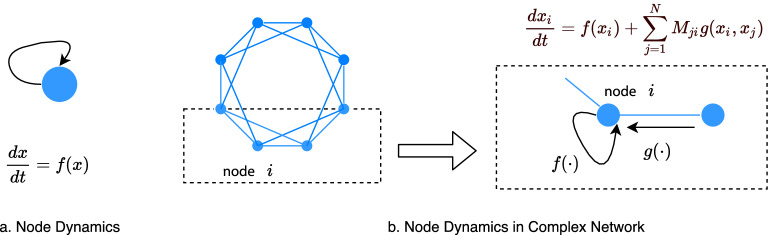
It shows the dynamics of one-dimensional system and multi-dimensional system. In a one-dimensional system, node behavior is controlled only by the self-dynamics $f(\cdot )$. (**b**) In a multi-dimensional dynamic system, node behavior is characterized by the self-dynamics $f(\cdot )$ and the coupling dynamics $g(\cdot )$.

## Problem formulation

### Nonlinear dynamics and resilience of single node

**Figure 2:**
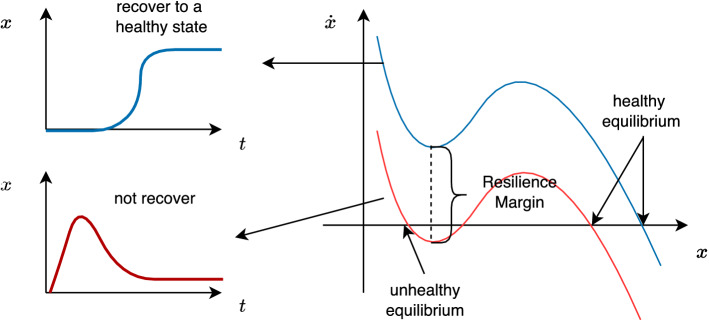
Red line describes a system with more than one stable equilibrium (healthy one and unhealthy one both exist). The system in this situation will remain in a low-level state. The blue line describes a system with only one stable equilibrium, and the system will recover to a high-level state at last. When there exists an unhealthy equilibrium, the system loses resilience. This picture shows the ability of the system to recover to the original state from an undesirable state. The resilience margin shows the ability of the system to withstand the perturbation. If the local minimum value is much larger than 0, the system can withstand more perturbations and keeps resilient. However, if the local minimum value is close to 0, small perturbation may cause the lose of resilience.

The behavior of a one-dimensional nonlinear dynamic system in ecology^32^, engineering^33^ etc. could be characterized by the equation:

1 $$\begin{aligned} \dot{x}=f(x), \end{aligned}$$

where *x* is the state of the system evolving with time (shown in Fig. 1a). One of the stable fixed points $x_0$ of Eq. (1) could be found by

2 $$\begin{aligned} f(x_0)=0 \end{aligned}$$

and

3 $$\begin{aligned} \left. \frac{df}{dx} \right| _{x=x_0}<0, \end{aligned}$$

where *f* is smooth, Eq. (2) provides the system’s steady state and Eq. (3) guarantees its linear stability. We assume that a stable equilibrium $x_e>0$ always exists which is away from the origin. Besides, the bifurcation may occur near to the origin. We define two different kinds of stable equilibrium: *healthy* equilibrium and *unhealthy* equilibrium. The *healthy* equilibrium is far from the origin and it is a desired state of the system. The *unhealthy* equilibrium is near to the origin and it is an undesired one. If there only exists the healthy equilibrium the system is resilient. While, if in the system healthy and unhealthy equilibria exist at the same time, the system will transit from the desired stable fixed point to the undesired one, which indicates the loss of resilience in the system shown in Fig. 2. Here we illustrate this concept by exploring the abundance of species in an ecological network^2^. If a healthy equilibrium and an unhealthy equilibrium exist, the system will transit to an unhealthy state, which means that the species is in an undesirable low-abundance state. In this situation, the system lose its resilience. However, if there only exist a healthy equilibrium away from origin, the system will maintain a high-abundance state. That is to say the system keeps resilient.

**Figure 3:**
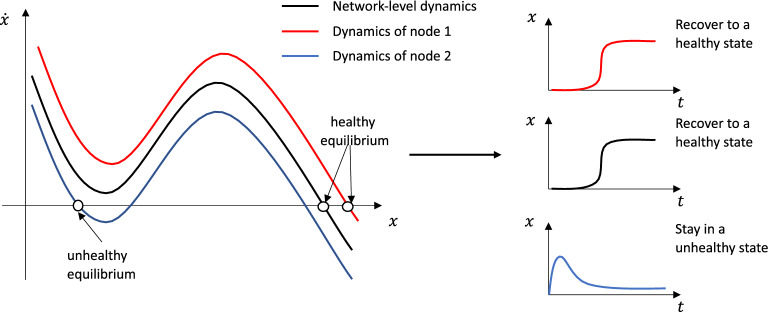
Similar network dynamics hide different node dynamics. It shows different dynamic response at node level. While the mean dynamics shows that the network is resilient, node 1 and node 2 have different dynamics.Node 1 only has one healthy equilibrium, but node 2 has a healthy equilibrium and an unhealthy equilibrium. Node 1 recovers to the healthy state, while node2 remains in an unhealthy state at last.

### Dynamics and resilience of system with connected nodes

Networked system often consists of a large number of components interacting with each other through the network shown in Fig. 1b. The dynamics of a node in the networked system of *N* nodes is defined by

4 $$\begin{aligned} \dot{x_i} = f(x_i, {\textbf {a}})+\sum _{j=1}^N {\textbf {M}}_{ji} g(x_i,x_j, {\textbf {b}}). \end{aligned}$$

Node *i*’s behavior is characterized by the self-dynamics $f(\cdot )$ and the coupling dynamics $g(\cdot )$. ${\textbf {M}}_{ji}$ is the element of connecting matrix ${\textbf {M}}$. ${\textbf {a}}$ and ${\textbf {b}}$ both are vectors of parameters. The number of parameters in ${\textbf {a}}$ and ${\textbf {b}}$ is $N_1$ and $N_2$, respectively. Here, we define vectors ${\textbf {x}}=(x_1, x_2\ldots , x_N), {\textbf {a}} =(a_1, a_2, \ldots , a_{N_1})$, ${\textbf {b}} = (b_1, b_2\ldots , b_{N_2})$.

Generally, the relationship between topology (e.g. properties of ${\textbf {M}}_{ji}$) and resilience of the network is still not very clear. One way to solve this problem is to compress the multi-dimensional dynamics to one-dimensional dynamics and map the overall effective dynamics of a networked system to its topology. Indeed, a common network-level effective dynamics may hide different node-level dynamics of different nodes (shown in Fig. 3). Also, the effects of uncertainty on macro scale and micro scale is unclear.

## Methods

**Figure 4:**
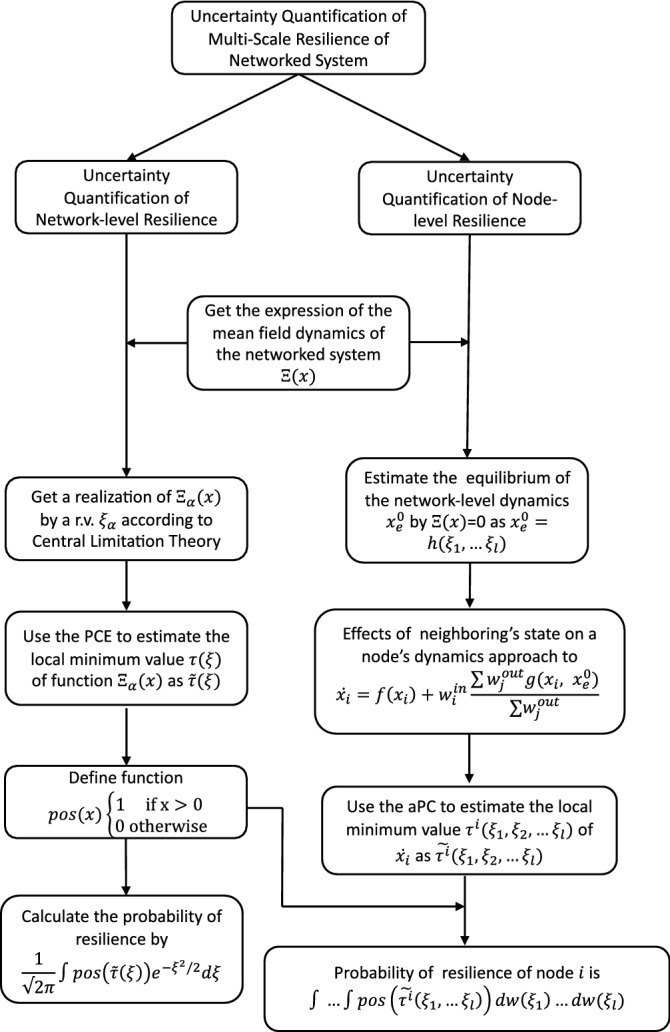
Method to quantify the uncertainty of multi-scale resilience of networked system.

To address this question, a specific sequential estimation method with aPC is used to estimate the effect of uncertainty on resilience at both network-level and node-level (The method is shown in Fig. 4). We do so by defining arbitrary uncertainty distributions on system’s parameters. A mean field estimation is used to approximate the network-level resilience and PCE is used to quantify the effect of uncertainty on network-level resilience as we have done in our previous work^15^. By this step, we can quantify the effect of uncertainty on network level.

**Figure 5:**
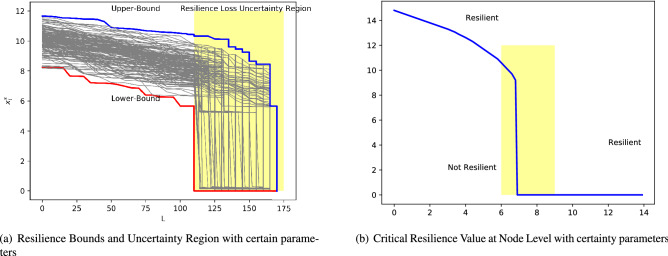
Critical resilience value identifies vulnerable nodes with certain parameters. (**a**) Resilience bounds show the upper-bound and lower-bound of equilibrium when links removed. In this figure, it explicitly predicts when the loss of resilience will happen. (**b**) Critical resilience shows the relationship between average weight value of network and critical weight value. When $w_i^\mathrm{in}>w_\mathrm{crit}$, the node is resilient; otherwise it is not.

To quantify the effect of uncertainty on node-level resilience, we first need to employ a specific sequential estimation method to estimate the node-level dynamics, and then use aPC to quantify uncertainty on each node. In Eq. (4), we know that the dynamics of a node in a networked system is determined by the self-dynamics function and the coupling dynamics function of neighboring nodes. The basic idea of the sequential estimation method is to estimate the effects on each node’s dynamics from network-level state to neighboring nodes’ state. In the sequential estimation method, a mean-field estimation is firstly used to approximate the network-level state. Then, the previous approximation can be used to estimate the effect of neighboring nodes’ state on a single node dynamics to get more accurate estimation of node-level dynamics. Finally, aPC is used to quantify the uncertainty on node-level resilience.

### Network-level resilience with uncertainty

Uncertainty may exist in self-dynamics, coupling dynamics, or topology of networked systems. It is assumed that uncertain parameters in dynamic functions could be represented by random variables. Parameters may obey distribution like Gaussian distribution, Beta distribution, etc., or we only know the PDF of parameters. Firstly, we could estimate the equilibrium of the mean-field dynamics of the networked system, which can be calculated by

5 $$\begin{aligned} \Xi (x) := \frac{1}{N}\sum _{i=1}^N(f(x,{\textbf {a}}))+\frac{1}{N}\sum _{i,j=1}^N {\textbf {M}}_{ji}g(x,x, {\textbf {b}}). \end{aligned}$$

$\Xi (x)$ depends on **a** and **b** which are both vectors of r.v.. Therefore, $\Xi (x)$ is a function depending on the random variable *x*. For fixed *x*, $f(x, {\textbf {a}})$ is a function depending on iid r.v. in ${\textbf {a}}$. We set

6 $$\begin{aligned} {\begin{matrix} &{}\mu _{f(x)}:=\mathbb {E}[f(x, {\textbf {a}})],\\ &{}\delta _{f(x)}:=\sqrt{{\textbf {Var}}[f(x, {\textbf {a}})]}.\\ \end{matrix}} \end{aligned}$$

Assume that the total number of uncertain parameters in vector ${\textbf {a}}$ and ${\textbf {b}}$ is *l*. Futher, we assume that the uncertain parameters in vector ${\textbf {a}}$ are represented by r.v. $\xi _1, \xi _2, \ldots \xi _{l_1}$ and uncertain parameters in vector ${\textbf {b}}$ are represented by r.v. $\xi _{l_{1+1}}, \xi _{l_{1+2}}, \ldots \xi _l$. $\mu _{f(x)}$ and $\delta _{f(x)}$ can be calculated by

7 $$\begin{aligned} \mu _{f(x)}=\int \ldots \int f(x, {\textbf {a}})dw(\xi _1)\ldots dw(\xi _{l_1}), \end{aligned}$$

8 $$\begin{aligned} \delta ^2_{f(x)}=\int \ldots \int (f(x,{\textbf {a}})^2-\mu _{f(x)}^2)dw(\xi _{1})\ldots dw(\xi _{l_1}), \end{aligned}$$

where $w(\xi _{i})$ represents the PDF of $\xi _{i}$.

According to Central Limit Theorem, for big enough *N* ($N\ge 30$), $\frac{1}{N}\sum _{i=1}^Nf(x, {\textbf {a}})$ can be approximated by a normally distributed random variable with mean $\mu _{f(x)}$ and standard deviation $\frac{1}{N}\delta _{f(x)}$, i.e.

9 $$\begin{aligned} \frac{1}{N}\sum _{i=1}^Nf(x, {\textbf {a}})\sim \mathscr {N}\left( \mu _{f(x)}, \frac{1}{N}\delta _{f(x)}^2\right) . \end{aligned}$$

$g(x,x,{\textbf {b}})$ is a function depending on random variable *x*. Similarly, we can get $\mu _{g(x)}$ and $\delta _{g(x)}$. Then we can get

10 $$\begin{aligned} \frac{1}{N}\sum _{i,j=1}^N{\textbf {M}}_{ji}g(x,x,{\textbf {b}})\sim \mathscr {N}\left( \frac{m}{N}\mu _{g(x)},\frac{m}{N^2}\delta _{g(x)}^2\right) , \end{aligned}$$

where $m=\sum _{i,j=1}^N{\textbf {M}}_{ji}$. From the above mentioned, we know that $\Xi (x)$ is the sum of 2 normally distributed r.v.. Then we have

11 $$\begin{aligned} \Xi (x)\sim \mathscr {N}\left( \mu _{f(x)}+\frac{m}{N}\mu _{g(x)},\frac{1}{N}\delta _{f(x)}^2+\frac{m}{N^2}\delta _{g(x)}^2\right) . \end{aligned}$$

$\Xi _\alpha (x)$ could be realised by drawing $\zeta _\alpha$ from $\mathscr {N}(0,1)$ and we can get

12 $$\begin{aligned} \Xi _\alpha (x) = \mu _{f(x)}+\frac{m}{N}\mu _{g(x)}+\sqrt{\frac{1}{N}\delta _{f(x)}^2+\frac{m}{N^2}\delta _{g(x)}^2}\zeta _\alpha . \end{aligned}$$

It is assumed that every realization of $\Xi (x)$ has the shape described in Fig. 3. To identify system’s resilience, the local minimum value $\tau$ can be used as an indicator. As is shown in Fig. 2, for a given realization of $\zeta _\alpha$, if $\tau _\alpha > 0$, there only exsits one healthy equilibrium and the system is resilient. If $\tau _\alpha < 0$, both the healthy equilibrium and unhealthy equilibrium exist. Therefore, the system loses resilience. The probability of the system to be resilient is ${\textbf {P}}(\tau >0)$. Since $\tau$ is a function of $\zeta$, $\tau (\zeta )$ can be estimated by PCE truncated to degree *r* and it is denoted by $\tilde{\tau }_r(\zeta )$ (the detail of PCE is shown in supplement material S1). Here, we define the function

13 $$\begin{aligned} pos(x) = \left\{ \begin{array}{lr} 1 \quad if \quad x>0 &{} \\ 0 \quad otherwise &{} \end{array}. \right. \end{aligned}$$

Since $\zeta _\alpha$ is a normal distributed r.v. in Eq. (12), then the probability of network-level resilience is

14 $$\begin{aligned} \frac{1}{\sqrt{2\pi }}\int pos(\tilde{\tau }_r(\zeta ))e^{-\zeta ^2/2}d\zeta . \end{aligned}$$

### Node-level resilience with uncertainty

From the above analysis, we can quantify the effects of uncertainty on network-level resilience. However, such effects on node-level resilience are still unclear. Here, a specific sequential estimation method is employed to estimate the node-level dynamics, and then the aPC is applied to quantify the uncertainty on each node.

#### Step 1

A mean field estimation is used to drive sequential substitution and estimation of node-level resilience. The initial estimation of equilibrium $x_e^0$ is calculated by $\Xi (x) := \frac{1}{N}\sum _{i=1}^N(f(x,{\textbf {a}}))+\frac{1}{N}\sum _{i,j=1}^N{\textbf {M}}_{ji}g(x,x, {\textbf {b}})=0.$ For a given connecting matrix ${\textbf {M}}$, $x_e^0$ is a function of uncertain parameters $\xi _1, \xi _2, \ldots , \xi _l$ in ${\textbf {a}}$ and ${\textbf {b}}$ and we set $x_e^0=h(\xi _1, \xi _2, \ldots , \xi _l)$. Notice that the method in the above section (Network-level resilience with Uncertainty) is not suitable to estimate the $x_e^0$ here. This is because according to Eq. (12), the estimation of equilibrium $x_e^0$ depends on the variable $\zeta$. $\zeta$ depends on uncertain parameters $\xi _1, \xi _2, \ldots \xi _l$. When we estimate the node-level dynamics by $x_e^0$, the estimation function will contain $\xi _1, \xi _2, \ldots \xi _l$ and $\zeta$, which are not independent with each other. This will make the uncertainty quantification of node-level resilience more complicated. Taking this into account, we use the function $h(\xi _1, \xi _2, \ldots , \xi _l)$ to represent $x_e^0$, which will not bring new variables when we estimate the node-level equilibrium.

#### Step 2

The previous approximation could be used to estimate the dynamics of the network-level state. In a networked system, the state of each node is affected by the state of its immediate neighbors. Therefore, in this step, we will further estimate the effects of neighboring nodes on a single node’s dynamics to estimate the equilibrium of a single node. The effect of an in-edge on the dynamics of node *i* is $g(\cdot )$ and the probability of a node *j* is on the other side of the in-edge is proportional to its out-degree. So, the average effect is $\sum _{j=1}^Nw_j^\text {out}g(x_i,x_j,{\textbf {b}})/\sum _{j=1}^Nw_j^\text {out}$. To approximate mean effect of the neighbors, components in $g(\cdot )$ are weighted by $w^\text {out}$. Therefore, the previous step’s estimation could be used to make further estimation. We can approximate the effects of neighboring nodes on single node’s dynamics by

15 $$\begin{aligned} \dot{x_i}=f(x_i, {\textbf {a}})+w_i^\mathrm{in}\frac{\sum _{j=1}^Nw_j^\text {out}g(x_i,x_{e}^0, {\textbf {b}})}{\sum _{j=1}^Nw_j^\text {out}}. \end{aligned}$$

As we have mentioned before that if there only exists one equilibrium in Eq. (15), node *i* is resilient. Otherwise, the node loses its resilience. To identify the resilience of node *i*, the local minimum value $\tau ^{i}$ of $\dot{x}_i$ can be used as an indicator. We have assumed that the uncertain parameters in ${\textbf {a}}$ and ${\textbf {b}}$ were represented by r.v. $\xi _1, \xi _2,\ldots ,\xi _l$. For a given realization of $\xi _1^{\alpha }, \xi _2^{\alpha },\ldots ,\xi _l^{\alpha }$, if $\tau _{\alpha }^{i}>0$, node *i* is resilient. The probability of a node to be resilient is ${\textbf {P}}(\tau ^i>0)$. For node *i*, $w_i^\mathrm{in}$ is determinated and $\tau ^{i}$ is a function of $\xi _1, \xi _2, \ldots ,\xi _l$. $\tau ^{i}(\xi _1, \xi _2,\ldots ,\xi _l)$ can be estimated by aPC, which is denoted by $\tilde{\tau }^i(\xi _1, \xi _2, \ldots ,\xi _l)$. Then, the probability of node *i* being resilient can be calculated by

16 $$\begin{aligned} \int \ldots \int _lpos(\tilde{\tau }^i(\xi _1,\ldots ,\xi _l))dw(\xi _1)\ldots dw(\xi _l). \end{aligned}$$

The indicator $\tau ^{i}(\xi _1, \xi _2, \ldots , \xi _l)$ of node *i* can be approximated by a multivariate polynomial expansion

17 $$\begin{aligned} \tau ^i(\xi _1, \xi _2, \ldots , \xi _l)=\sum _{i=1}^Zc_i\Phi _i(\xi _1, \xi _2, \ldots , \xi _l). \end{aligned}$$

The number of *Z* in Eq. (17) is decided by the number of input parameters *l* and the expansion order *r* according to the formula^27^

18 $$\begin{aligned} Z = (l+r)!/(l!r!). \end{aligned}$$

Here, we need to construct the orthogonal polynomial basis $\Phi _i$ for $\xi _1, \xi _2,\ldots ,\xi _l$. Assuming that the input parameters are independent, the multi-dimensional basis can be constructed as a simple product of the corresponding univariate polynomials

19 $$\begin{aligned} {\begin{matrix} &{}\Phi _i(\xi _1, \xi _2, \ldots , \xi _l)=\prod _{j=1}^lP_j^{(\alpha _j^i)}(\xi _1, \xi _2,\ldots ,\xi _l), \\ &{}\sum _{j=1}^l\alpha _j^i\le Z, \quad i=1,\ldots ,l. \end{matrix}} \end{aligned}$$

In Eq. (19), $\alpha _j^i$ is a multivariate indicator with information on how to list all possible products of individual univariate basis functions. We define the polynomial $P^{(k)}_j(\xi _j)$ of degree *k* in the random variable $\xi _j$ as

20 $$\begin{aligned} P_j^{(k)}(\xi _j)=\sum _{i=0}^kP_{i,j}^{(k)}\xi _{j}^i, k \in \left[ 0, r\right] , \end{aligned}$$

where $P_{i,j}^{(k)}$ are coefficients in $P_j^{(k)}(\xi _j)$. The key of the aPC method is to construct the polynomials in Eq. (20) to form an orthonormal basis for arbitrary distributions which could be discrete, continuous raw data sets or by their moments. We define the orthonormality for polynomials $P^{(k)}_j$ and $P_j^{(q)}$ as

21 $$\begin{aligned} \int P^{(k)}_j(\xi _j)P^{(q)}_j(\xi _j)dw(\xi )= \left\{ \begin{array}{lr} 0 \quad \forall k\ne q &{} \\ 1 \quad else &{} \end{array}. \right. \end{aligned}$$

Here we assume the leading coefficients of all polynomials: $P_{k,j}^{(k)}=1 \quad \forall k$. The *k*th raw (crude) moment of the random variable is defined as

22 $$\begin{aligned} \mu _{k,j} = \int \xi _j^kdw(\xi _j). \end{aligned}$$

The relationship between raw moments of $\xi _j$ and their coefficients $P_{i,j}^{(k)}$ can be written in matrix form (the detail process could be seen in^27,34^)

23 $$\begin{aligned} \left[ \begin{array}{cccc} \mu _{0, j} &{} \mu _{1, j} &{}\cdots &{} \mu _{k,j}\\ \mu _{1, j} &{} \mu _{2, j} &{}\cdots &{} \mu _{k+1, j}\\ \vdots &{}\vdots &{}\vdots &{} \vdots \\ \mu _{k-1, j} &{} \mu _{k, j} &{}\cdots &{} \mu _{2k-1, j}\\ 0 &{} 0 &{} \cdots &{} 1 \end{array} \right] \left[ \begin{array}{c} P_{0,j}^{(k)} \\ P_{1,j}^{(k)} \\ \vdots \\ P_{k-1,j}^{(k)}\\ P_{k, j}^{(k)} \end{array} \right] = \left[ \begin{array}{c} 0 \\ 0 \\ \vdots \\ 0\\ 1 \end{array} \right] . \end{aligned}$$

### Other types of uncertainty

Uncertainty mainly includes parameter uncertainty, model uncertainty, observation uncertainty, measure noise^35–37^. We have studied the effect of parameter uncertainty on resilience and now we discuss how observation uncertainty affects the resilience of the system. Firstly, we consider the observation noise

24 $$\begin{aligned} \dot{x_i} = f(x_i)+\sum _{j=1}^N {\textbf {M}}_{ji} g(x_i,x_j)+\gamma _i, \end{aligned}$$

where $\gamma _i$ is the observation uncertainty which can be regarded as a random variable. The mean-field dynamics of the networked system can be calculated by

25 $$\begin{aligned} \Xi (x) := \frac{1}{N}\sum _{i=1}^N(f(x))+\frac{1}{N}\sum _{i,j=1}^N {\textbf {M}}_{ji}g(x,x)+\frac{1}{N}\sum _{i=1}^N\gamma _i. \end{aligned}$$

The resilience of the mean-field dynamics can also be determined by the local minimum value. Here we set the local minimum value of the deterministic part $\frac{1}{N}\sum _{i=1}^N(f(x))+\frac{1}{N}\sum _{i,j=1}^N {\textbf {M}}_{ji}g(x,x)$ as $\tau _1$. If $\tau _1+\frac{1}{N}\sum _{i=1}^N\gamma _i>0$, the system is resilient. According to Central Limit Theorem, for big enough $N(30\le N)$, $\frac{1}{N}\sum _{i=1}^N\gamma _i$ can be approximated by a normally distributed random variable with mean $\mu _{\gamma }$ and standard deviation $\frac{1}{N}\delta _{\gamma }$, i.e.

26 $$\begin{aligned} \frac{1}{N}\sum _{i=1}^N\gamma _i\sim \mathscr {N}(\mu _{\gamma }, \frac{1}{N}\delta ^2_{\gamma }), \end{aligned}$$

where $\mu _{\gamma }:=\mathbb {E}[\gamma ]$ and $\delta _{\mu }:=\sqrt{{\textbf {Var}}[\gamma ]}$. Then $\frac{1}{N}\sum _{i=1}^N\gamma _i=\mu _{\gamma }+\sqrt{1/N}\delta _{\gamma }\zeta$, where $\zeta$ is a normal distributed random variable. If $\tau _1+\mu _{\gamma }+\sqrt{1/N}\delta _{\gamma }\zeta >0$, the system is resilient, which equals to $\zeta >-\frac{(\tau _1+\mu _{\gamma })}{\sqrt{1/N}\delta _{\gamma }}$. The probability to be network-level resilient is

27 $$\begin{aligned} \frac{1}{2\pi }\int ^{+\infty }_{-\frac{(\tau _1+\mu _{\gamma })}{\sqrt{1/N}\delta _{\gamma }}} e^{-\zeta ^2/2}d\zeta . \end{aligned}$$

To quantify the effect of uncertainty on the node-level resilience, according to our method, firstly we need to calculate the equilibrium $x_e^0$ of the mean-field dynamics by

28 $$\begin{aligned} \Xi (x) := \frac{1}{N}\sum _{i=1}^N(f(x))+\frac{1}{N}\sum _{i,j=1}^N {\textbf {M}}_{ji}g(x,x)+\frac{1}{N}\sum _{i=1}^N\gamma _i=0. \end{aligned}$$

We can approximate the effects of neighboring nodes on single node’s dynamics by

29 $$\begin{aligned} \dot{x_i}=f(x_i)+w_i^\mathrm{in}\frac{\sum _{j=1}^Nw_j^\text {out}g(x_i,x_{e}^0)}{\sum _{j=1}^Nw_j^\text {out}}+\gamma _i. \end{aligned}$$

The local minimum value of the deterministic part $f(x_i)+w_i^\mathrm{in}\frac{\sum _{j=1}^Nw_j^\text {out}g(x_i,x_{e}^0)}{\sum _{j=1}^Nw_j^\text {out}}$ is set as $\tau _2$. If $\tau _2+\gamma _i>0$, then node *i* is resilient. The probability of node *i* to be resilient is $\int _{\tau _2}^{+\infty }w(\gamma )d\gamma$, where $w(\gamma )$ is the probability density function of $\gamma$.

**Figure 6:**
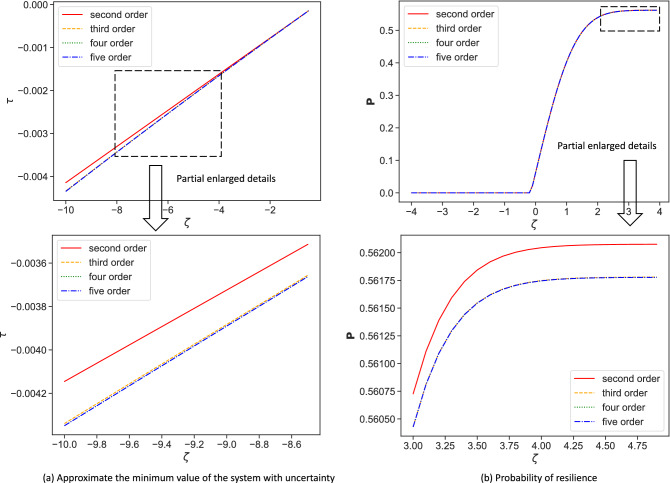
Approximate resilience of system by Polynomial Chaos Expansion. We truncate the series to order *r* from 2 to 5. (**a**) Approximate the minimum value of the system by PCE. (**b**) Approximate the probability of resilience. It is clear that there is a significant difference in results between $r=2$ and $r=3,4,5$ in (**a**) and (**b**).

**Figure 7:**
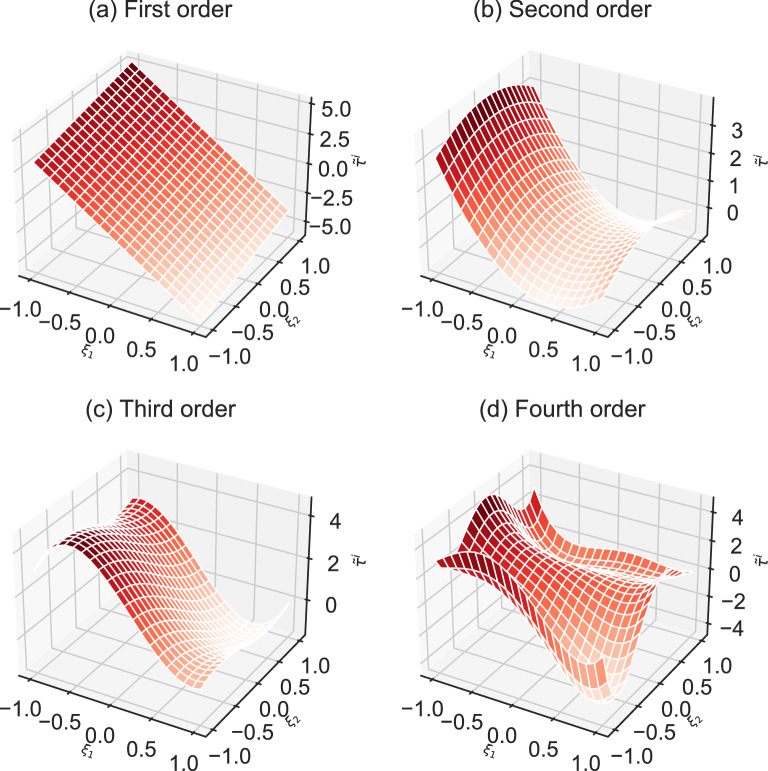
Approximate the local minimum value $\tau ^i$ of node *i* by aPC. These four subfigures show the results of aPC truncate to different orders from 1 to 4. If $\tau ^i>0$, node *i* is resilient. Otherwise, the node loses resilience.

## Results

Bi-stable dynamical systems are common across social (e.g., population logistic model^38^), ecological (e.g., soil health^39^), climate (e.g., ocean circulation^40^), and human conflict systems^41^. There exist a stable undesirable state (e.g., population collapse or conflict) and a stable desirable state (e.g., healthy population with collaboration^42^), with an unstable transition brink in between, and this is ideal for demonstrating the concept of resilience and uncertainty. Networks that connect such systems represent a wider interacting ecosystem and often a mutualistic coupling represents positive reinforcing interactions. Interaction examples include gravity, radiation, or Boltzmann Lotka Volterra (BLV) models^43^ frequently use a $x_i \times x_j$ mutualistic attractor component.

### Case study A: ecological pollinator network

A case of pollinator networks^44^ is used to illustrate the dynamics of networked system at micro and macro scale. $x_i$ represents the abundance of species *i*, which is given by

30 $$\begin{aligned} \frac{dx_i}{dt}=B_i+x_i\left( 1-\frac{x_i}{K_i}\right) \left( \frac{x_i}{C_i}-1\right) +\sum _{j=1}^N {\textbf {M}}_{ji}\frac{x_ix_j}{D_i+E_ix_i+H_jx_j}. \end{aligned}$$

$B_i$ represents the incoming migration rate of species *i* from other ecosystems. The second term on right hand shows logistic growth with carrying capacity $K_i$ of the system, and the Allee effect (low abundance ($x_i<C_i$) causes negative growth)^45^. The third term is a coupling function which saturates for large $x_i$ or $x_j$ (*j*’s positive contribution to $x_i$ is bounded).

For simplicity, we use homogeneous parameters: $B=0.1, C=1, K=5, D=5, E=0.9, H=0.1$. Besides, it is assumed that some parameters’ value has to be within $10\%$ of its mean. Here, we set $C = \mathbb {E}[C](1+0.1\xi _1), E=\mathbb {E}[E](1+0.1\xi _2)$, where $\xi _1, \xi _2$ are random variables uniform in $[-1, 1]$ ($\xi _1, \xi _2$ could be r.v. that follow arbitrary distributions.). The definition of system resilience in this model is the ability of the system to recover species abundance from extinction^12^. To achieve this, the system should be in the regime where only one equilibrium exists. This is because if over one equilibrium exists in the system, the system will be trapped in the state with low abundance, which means that the system cannot recover its species abundance and loses its resilience.

#### Relationship between network topology and resilience

In Fig. 5, we show what happens when a network becomes less connected by removing edges. In this case, parameters are certain and the figure explicitly shows the bounds of equilibrium under different perturbations and the regime where loss of resilience happens. Critical function describes resilience regime which maps macro (network-level) properties (average weighted degree $w_\text {av}$ to micro (local-level) properties (critical resilience value $w_\text {crit}$), $w_\mathrm{av}=\frac{1}{N}\sum _{i=1}^N\sum _{j=1}^N{\textbf {M}}_{ji}$). For each $w_\text {av}$, corresponding $w_\text {crit}$ could be calculated by

31 $$\begin{aligned} \dot{x_i} = f(x_i)+w_i^\mathrm{in}g(x_i, x_e^0(w_\text {av})). \end{aligned}$$

The critical weight, $w_\text {crit}$, is a function of $w_\text {av}$ since it is a function of $x_e^0$ and $x_e^0$ is a function of $w_\text {av}$. In Fig. 5b, we see the graph of $w_\text {crit}$ versus $w_\text {av}$. Since $x_e^0$ is discontinuous, $w_\text {crit}$ is also discontinuous.

In this case, a threshold value of critical weight $w_{*}$ is about 6.9 where the bifurcation will happen. When the average weight is greater than 6.9, the system is resilient and almost every node in this system is resilient. The critical weight can reveal some basic properties for the dynamics on a nodal level. For example, we see in Fig. 5b that when when $w_\text {av}>w_{*}$, $w_\text {crit}$ is almost 0. This implies that if the system on average is in the resilient region, a node will also be in the resilient region even if it is very weakly connected to the rest of the network. However, in the case with uncertain parameters, even if the average weight is greater than 6.9, the system is possibly not resilient.

**Table 2:** Methods to estimate node-level resilience.

Method	Truncate order/Number of samples	Accuracy	Computational time (s)	Explainability
Specific sequential estimation method with aPC	1	0.5233	4.58	Yes
	2	0.8224	4.89	
	3	0.9495	5.84	
	4	0.9502	6.11	
Specific sequential estimation methodwith Monte Carlo	50	0.7549	7.9	No
	100	0.9299	10.9	
	400	0.9834	27.2	
	1000	0.991	68.79	

**Figure 8:**
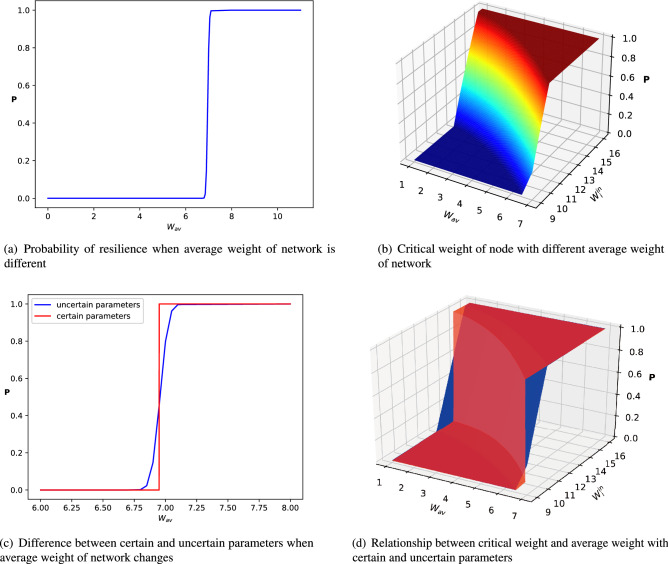
It shows the effect of uncertainty parameters on resilience of network and each node. (**a**,**b**) The probability of resilience at network-level and node-level in system with uncertainty. (**c**,**d**) The difference between resilience with certain parameters and uncertain parameters at network-level and node-level (blue represents system with uncertain parameters and red represents system with certain parameters).

**Figure 9:**
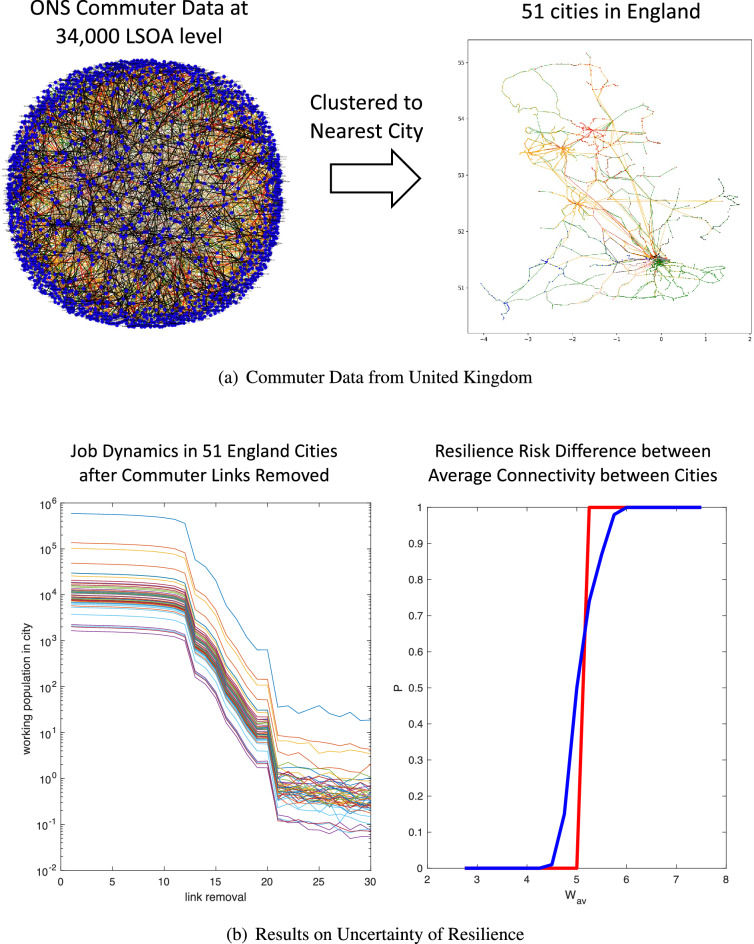
UK commuter network with urban job dynamics coupled by a competitive commuter mobility model. (**a**) Raw data consists of census data of home to work travel across 34,000 Lower Layer Super Output Areas (LSOA). This is then clustered to nearest major cities and the number of services for commuting inform the weight of the matrix. (**b**) simulated labor mobility dynamics with transport link removal causes simulated mobility dynamics to collapse.

#### Analysis on the effect of uncertainty

We initially do not know what the exact order *r* is for an accurate estimation. For a given problem, it is not trivial to analytically find the optimal *r*. Usually, this is done heuristically. We truncate the series to arbitrary orders *r* from 2 to 5 (Fig. 6). It is clear that the convergence of the function can be improved with the increase of the polynomial order *r*. However, with the increase of the order, much more simulation is needed. Therefore, we have to make a compromise between accuracy and computational efficiency. In Fig. 6, it clearly shows the difference among different orders, especially $d=2$. To calculate the probability of resilience, a graph of Cumulative Distribution Function (CDF) with different truncation is shown in Fig. 6b. In Fig. 6, when $d=3, 4, 5$, the results are almost the same. However, there is an obvious difference for $d=2$. Considering the accuracy and computational efficiency, we choose $d=3$ for the polynomial order. So, we can see the effect of uncertain parameters on system resilience as well as node-level resilience. When parameters are certain and the average weight is 6.9, the system is resilient and all nodes are resilient. However, when parameters are uncertain in this case, the probability of resilience of the system is about 0.561. So according to the analysis above, some nodes will also possibly lose resilience.

Secondly, we use specific sequential estimation method with aPC to estimate the resilience of each node. We truncate the series to arbitrary orders *r* from 1 to 4 shown in Fig. 7 to estimate the resilience indicator $\tau ^i$. The probability of node *i* to be resilient is ${\textbf {P}}(\tau ^i>0)$. Since we get the polynomial chaos expansion with different truncate order, according to Eq. (16), we can calculate the probability to be resilient. The results are respectively 0.783, 0.625, 0.54, 0.5396. To check the accuracy of aPC with different truncate order, the result estimated by Monte Carlo with 2000 samples is regard as the standard (The probability of node resilience is 0.514). Table 2 shows the results of specific sequential estimation method with aPC and Monte Carlo. With the increase of truncate order, the accuracy of aPC improves a lot without much increase of computational time. Otherwise, the computational time of specific sequential estimation method with Monte Carlo increases a lot with the increase of samples. Besides, the specific sequential estimation method with aPC provides the analytic expression of resilience with uncertainty which helps to explain the effects of uncertainty on node-level resilience. However, the Monte Carlo methods lack explainability. Considering the accuracy, computational time, and explainability, the specific sequential estimation method with aPC is effective to estimate the resilience of individual nodes.

In Fig. 8, we show effects of uncertainty on the resilience of the whole network and each node. In Fig. 8a, it is clear that with certain parameters the networked system could maintain its resilience when the average weight is greater than 6.9. However, with the effect of uncertain parameters, the system could lose resilience even though its average weight is greater than 6.9. With the growth of average weight, the system has more chance to be resilient. When the average weight is greater than a certain value, the system is absolutely resilient. Similarly, in Fig. 8d red part shows that when node’s weight is greater than a critical value under a certain average weight, the node could maintain its resilience. While, with the effect of uncertainty represented by blue part, a node may lose resilience even though its weight is greater than the critical value in Fig. 5b. Therefore, the method mentioned above could help us understand the effect of uncertainty on network-level and node-level resilience. Also, it help us to predict whether a node is resilient and the probability of a node to lose resilience.

### Case study B: work force commuter network

#### Introduction to commuter networks

In our second example, we are motivated by a commuter network, where census data is used to populate how UK commuters travel from home to work (and vice versa). Here, we focus on the rail network as per earlier study by ourselves published in^46^. Each node is a railway station in the UK closest to the person’s home or work address (and that has indicated they use rail to travel to work) - all data can be found in^46^. From previous studies, we know that the well-established case of Boltzmann-Lotka-Volterra dynamics (similar to gravity and radiation mobility models) can be used to model the commuter competition dynamics^43^. Here, $x_i$ represents the total number of workers in city *i*, which is given by

32 $$\begin{aligned} \frac{dx_i}{dt}=x_i\left( 1-\frac{x_i}{K_i}\right) \left( \frac{x_i}{C_i}-1\right) + \sum _{j=1}^N {\textbf {M}}_{ji} \frac{x_j \exp [\alpha \log (x_i)-d_{ij}^{\beta }]}{\sum _{k}^N M_{jk} x_k \exp [\alpha \log (x_i)-d_{ik}^{\beta }]}. \end{aligned}$$

The first term on right hand shows logistic growth with transport carrying capacity $K_i$ of the system, and the Allee effect (e.g., the city will collapse if less than $C_i$ jobs are available). The second term is a coupling competition function which shows that the number of people coming to city *i* from city *j* is also subject to the competitive dynamics of other neighboring connected cities *k* in the denominator. The parameters of: $\alpha$ signify the general attractiveness of cities that already have a lot of jobs (critical mass effect), *d* signify the distance between cities and $\beta$ signify the impact of distance as a cost.

#### Data and analysis

We used the last census data from England in the United Kingdom (Office of National Statistics) to weigh the work force population parameter $x_i$ given above. The parameters are *C* is assumed to be a minimum 10% of jobs per city to maintain criticality, $K=$ jobs per city at census, $\alpha =0.5, \beta =2$ are standard scaling components, and the data is mapped to a real map of the United Kingdom using rail as the main source of inter-city links to inform ${\textbf {M}}_{ji}$, which we show the uncertainty impact of this assumption below.

The results (in a similar manner to Case Study A) are shown in Fig. 9. Part (a) shows our data acquisition of commute travel from different postcode areas in England and then clustering this to 51 major cities and linking them by major rail transport as a demonstration (see^46^ for more details of this process and we acknowledge the authors and data sources there). Part (b), we show that transport link removal is impacting the ability for jobs to be satisfied in cities and how uncertainty in the rail as only source of link between cities causes uncertainty fluctuations in resilience probability. As in our previous work^46^, a short discussion on the impact of this work is given here. Each link is actually a service, not necessarily a physical rail track. As such, link removal or a reduction in the average link weight means a reduction in services during the rush hours for commuting. What we show is that there is a criticality and this relates to specific links being more sensitive to others. As in our previous studies, we did focus on specific stations in the Thameslink for example, where the addition or removal of services can have a significant effect (see more in^46^). This did lead to a detailed discussion with Department for Transport to understand UK rail network resilience and the role of certain critical service links.

## Conclusion

Resilience is an important ability of a system to maintain its original function when perturbations happen. At present, the research of how to estimate resilience of dynamic networked systems with uncertainty is still limited. Node-level resilience is important to make critical interventions to specific components whilst preserving our multi-scale understanding of general system behavior. To solve this problem, a specific sequential estimation method with multi-dimensional arbitrary polynomial chaos (aPC) was employed in this paper to quantify the effects of uncertain parameters on macro and micro resilience. Besides, we have compared dynamics with certain parameters to dynamics with uncertainty when estimating the micro and macro resilience. This could help us make a prediction of macro and micro-scale behavior in networked systems and reduce the risk of uncertainty. However, how the community structure of a network affects network-level and node-level resilience is still vague, for example, whether there exists a relationship between the modularity of community in a network and resilience. Therefore, in the future, we will explore the effects of community structure of networks on dynamics.

## Supplementary Information

Supplementary Information.

## Data Availability

The dataset used in Case Study A: Ecological Pollinator Network is available at https://github.com/Martinezou/Ecological-Pollinator-Network.git. The dataset used in Case Study B: Work Force Commuter Network is available at https://www.ons.gov.uk/census/2011census.
