# Supplementary material for: Uncertainty quantification of multi-scale resilience in networked systems with nonlinear dynamics using arbitrary polynomial chaos
Source: Sci Rep. 2023 Jan 10;13:488. doi: 10.1038/s41598-022-27025-w (PMC9831990; doi:10.1038/s41598-022-27025-w)
Supplement: Supplementary file 1 — Supplementary Information. [file 41598_2022_27025_MOESM1_ESM.docx]

**Supplement Information**

**1.Date availbility**

The datasets used and/or analysed during the current study available from the corresponding author on reasonable request.

**2**. **Polynomial Chaos Expansion (PCE) Method**

Since $\tau$ is a function of $\zeta$, $\tau(\zeta)$ can be estimated by PCE and it is denoted by $\tilde{\tau}(\zeta)$. Let $w$ be the probability density function of $\zeta$. Our goal is to approximate $\tau(\zeta)$ by a polynomial series of $\zeta$. For this we need a family of polynomials $P_{n}$. Notice that $P_{0}$ is not $0$. For all $n$, the polynomials $n$ have degree $r$ and are orthogonal with respect to $w$, i.e., the inner product

$$<P_{n}, P_{m}>_{w}=\int_{-\infty}^{+\infty} P_{n}\left( \zeta\right)P_{m}\left( \zeta\right)w\left( \zeta\right)d\zeta$$

is $0$ when $m\neq n$ and the inner product is 1 when $m=n$. The polynomials $P_{n}$ can be used as a basis. So we can write

$\tilde{\tau}\left( \zeta\right)=\sum_{n=0} c_{n}P_{n}(\zeta)$.

In order to get the expression of $\tilde{\tau}\left( \zeta\right)$, we need to define the orthogonal basis $P_{n}$ and the coefficients $c_{n}$. What kind of orthogonal basis should be chosen depends on the distribution of random variable $\xi$. We have mentioned in our paper that $\zeta$ follows Gaussian distribution, so the Hermite polynomial can be chosen as the orthogonal basis. The different orders of Hermite polynomials are shown in the table S1.

In order to do any computation with a PCE series, we need to truncate it. First, we notice that if the series converges, then the size of each coefficient goes to 0 if we take the limit of any index to infinity. This means that for such convergent series we can ignore terms with order higher than some $r$. However, for a given problem, it is not trivial to find which exactly this $r$ is. Usually, this is done by trial and error, where we can calculate more terms until the size of the new terms is smaller than the precision we need.

For the computation of the coefficients, we will use a nonintrusive method. We start by truncating the series to an arbitrary order $r$, $\tilde{\tau}_{r}\left( \zeta\right)=\sum_{n=0}^{r} c_{n}H_{n}(\zeta)$ and assume that this is enough for the wanted precision. Then we generate $r_{1}>r$ instances of the random variable $\zeta$, $\{\zeta_{1},\zeta_{2},\ldots,\zeta_{r_{1}}\}$. Then for every $\zeta_{i}$ we have the equation

$$\tilde{\tau}_{r}\left( \zeta_{i} \right)=\sum_{n=0}^{r} c_{n}H_{n}(\zeta_{i})$$

Notice that for each $\zeta_{i}$, we can get the value of $\tau(\zeta_{i})$ and $H_{n}(\zeta_{i})$. So, we can compute the coefficients $c_{n}$ by solving a linear regression. After that we compute $\sup_{\zeta}\left| c_{n}P_{n}(\zeta) \right|$ and if it is smaller than the precision we stop, otherwise we increase $r$ and repeat the process.

**3.Supplement of Arbitrary Polynomial Chaos Expansion (aPC) Method**

a. One-dimensional aPC

It is assumed that the minimal value of the system $\tau$ is a function of one uncertain parameter $\xi$. $\tau(\xi)$ can be estimated by aPC, which is denoted by $\tilde{\tau}\left( \xi\right)$. $\tilde{\tau}\left( \xi\right)$ is expanded as a polynomial expansion as:

$\tilde{\tau}\left( \xi\right)=\sum_{i=1}^{Z} c_{i}P^{i}(\xi)$, (1)

where $Z$ is the order of expansion, $c_{i}$ are the expansion coefficients, and $P_{i}$ are the orthogonal polynomial basis. The polynomial basis can be constructed by

$P^{k}\left( \xi\right)=\sum_{i=1}^{k} p_{i}^{(k)}\xi^{i}, k\in[0, Z]$, (2)

where $p_{i}^{(k)}$ are coefficients in $P^{k}\left( \xi\right)$. The key of aPC method is to construct the polynomials in equation (2) to form an orthonormal basis for arbitrary distributions which could be discrete, continuous raw data sets or by their moments. We define the orthonormality for polynomials $P^{(k)}$ and $P^{(l)}$ as

$\int P^{l}\left( \xi\right)P^{k}(\xi)dw(\xi)=\left\{ \begin{aligned} 0 \forall k\neq l \\ 1 \mathrm{else} \end{aligned} \right.$, (3)

where $w(\xi)$ is the probability density function of $\xi$. Firstly, an intermedia auxiliary condition can be introduced by demanding that leading coefficients of all polynomials equals to 1. $p_{k}^{(k)}=1 \forall k$. We can obtain an orthogonal basis by

$\int p_{0}^{(0)}\left[ \sum_{i=0}^{k} p_{i}^{\left( k \right)}\xi^{i} \right]dw\left( \xi\right)=0$,

$$\vdots$$

$$\int\left[ \sum_{i=0}^{k-1} p_{i}^{\left( k-1 \right)}\xi^{i} \right]\left[ \sum_{i=0}^{k} p_{i}^{\left( k \right)}\xi^{i} \right]dw\left( \xi\right)=0,$$

$p_{k}^{(k)}=1$. (4)

Since the $k$th raw moments of $\xi$ can be calculated by $\mu_{k}=\int\xi^{k}dw(\xi)$. Equation (4) equals to

$\sum_{i=0}^{k} p_{i}^{(k)}\mu_{i}=0$,

$$\vdots$$

$\sum_{i=0}^{k} p_{i}^{(k)}\mu_{i+k-1}=0$,

$p_{k}^{(k)}=1$. (5)

It is more convinient to determine $p_{i}^{(k)}$ by the matrix form:

$\left[ \begin{matrix} \mu_{0} & \mu_{1} & \ldots& \mu_{k} \\ \mu_{1} & \mu_{2} & \ldots& \mu_{k+1} \\ \vdots& \vdots& \vdots& \vdots\\ \mu_{k-1} & \mu_{k} & \ldots& \mu_{2k-1} \\ 0 & 0 & \ldots& 1 \end{matrix} \right]\left[ \begin{matrix} p_{0}^{(k)} \\ p_{1}^{(k)} \\ \vdots\\ p_{k-1}^{(k)} \\ p_{k}^{(k)} \end{matrix} \right]=\left[ \begin{matrix} 0 \\ 0 \\ \vdots\\ 0 \\ 1 \end{matrix} \right]$ (6)

b. Multi-dimensional aPC

It is assumed that the minimal value of the system $\tau$ is a function of uncertain parameters $\xi_{1}, \xi_{2},\ldots,\xi_{l}$. Then $\tau(\xi_{1}, \xi_{2},\ldots\xi_{l})$ can be approximated by a multivariate polynomial expansion

$\tilde{\tau}=\sum_{i=1}^{Z} c_{i}\Phi_{i}\left( \xi_{1}, \xi_{2},\ldots,\xi_{l} \right)$. (7)

The number of $Z$ in equation (7) is decided by the number of input parameters $l$ and the expansion order $r$ according to the formula $Z=\left( l+r \right)!/(l!r!)$. For example, if the number of uncertain parameters $l=2$ and expansion order $r=3$, $Z=\frac{\left( 2+3 \right)!}{2!3!}=10$. $l=2, r=4, Z=\frac{\left( 2+4 \right)!}{2!4!}=15$. Table S4 shows the number $Z$ of terms in polynomial expansion with different expansion order and variable number. The multi-dimensional basis can be constructed as a simple product of the corresponding univariate polynomials

$$\Phi_{i}\left( \xi_{1},\xi_{2},\ldots,\xi_{l} \right)=\prod_{j=1}^{l} P_{j}^{\left( \alpha_{j}^{i} \right)}(\xi_{1}, \xi_{2},\ldots,\xi_{l}),$$

$\sum_{j=1}^{l} \alpha_{j}^{i}\leq Z, i=1,2\ldots,l$. (8)

In equation (8), $\alpha_{j}^{i}$ is a multivariate indicator with information on how to list all possible products of individual univariate basis functions. We define the polynomial $P_{j}^{\left( k \right)}(\xi_{j})$ of degree $k$ in the random variable $\xi_{j}$ as

$P_{j}^{\left( k \right)}\left( \xi_{j} \right)=\sum_{i=0}^{k} p_{i,j}^{(k)}\xi_{j}^{i}, k\in[0, r]$, (9)

where $p_{i,j}^{(k)}$ are coefficients in $P_{j}^{\left( k \right)}(\xi_{j})$.

The key of the aPC method is to construct the polynomials in equation (9) to form an orthonormal basis for arbitrary distributions which could be discrete, continuous raw data sets or by their moments. We define the orthonormality for polynomials $P_{j}^{(k)}$ and $P_{j}^{(q)}$ as

$\int P_{j}^{\left( k \right)}\left( \xi_{j} \right)P_{j}^{\left( q \right)}(\xi_{j})dw(\xi_{j})=\left\{ \begin{aligned} 0 \forall k\neq q \\ 1 \mathrm{else} \end{aligned} \right.$ , (10)

Here we assume that the leading coefficients of all polynomials: $p_{k,j}^{(k)}=1 \forall k.$ The $k$th raw (crude) moment of the random variable is defined as $\mu_{k,j}=\int\xi_{j}^{k}dw(\xi_{j})$.

The relationship between raw moments of $\xi_{j}$ and their coefficients $p_{i,j}^{k}$ can be written in matrix form

$\left[ \begin{matrix} \mu_{0,j} & \mu_{1,j} & \ldots& \mu_{k,j} \\ \mu_{1,j} & \mu_{2,j} & \ldots& \mu_{k+1,j} \\ \vdots& \vdots& \vdots& \vdots\\ \mu_{k-1,j} & \mu_{k, j} & \ldots& \mu_{2k-1, j} \\ 0 & 0 & \ldots& 1 \end{matrix} \right]\left[ \begin{matrix} p_{0,j}^{(k)} \\ p_{1,j}^{(k)} \\ \vdots\\ p_{k-1,j}^{(k)} \\ p_{k,j}^{(k)} \end{matrix} \right]=\left[ \begin{matrix} 0 \\ 0 \\ \vdots\\ 0 \\ 1 \end{matrix} \right]$ (11)

**4.Supplement of Figures**

Figure S1. Dynamics of one-dimensional system and multi-dimensional system. (a) Node Dynamics. In a one-dimensional system, node behavior is controlled only by the self-dynamics $f(\cdot)$. (b) Node Dynamics in Complex Network. In a multi-dimensional dynamic system, the system consists of nodes interacting through the complex network. If two nodes connected by an edge, then they are neighbouring nodes and affect each other’s behaviors. The connecting matrix $\boldsymbol{M}$ shows the connecting relationship of the network. If there is an edge exists between node $i$ and node $j$, $M_{ji}=1$. Otherwise, $M_{ji}=0$. In an undirect network, $M_{ji}=M_{ij}$ and the connecting matrix is symmetric. Therefore, in a multi-dimensional dynamic system, a node’s behavior depends on its self-dynamics $f(\cdot)$ and the coupling dynamics $g\left( \cdot\right)$ of neighbouring nodes.

Figure S2. Bi-stable system. The equilibrium of the system  $\dot{x}=f(x)$ can be found by solving the equation $f\left( x_{0} \right)=0$. The stability of the equilibrium is guaranteed by the function $\left. \frac{df}{dx} \right|_{x=x_{0}}<0$. For example, considering the function $f\left( x \right)=x^{2}-1$, there exists two solutions $x_{0}=-1$ and $1$. $x_{0}=-1$ is the only stable equilibrium of the system, because $\left. \frac{df}{dx} \right|_{x=-1}<0$, while $\left. \frac{df}{dx} \right|_{x=1}>0$. Red line describes a system with more than one stable equilibrium (healthy one and unhealthy one both exist). The system in this situation will stay in a low-level state. Blue line describes a system with only one stable equilibrium, and the system will recover to a high-level state at last. When there exists an unhealthy equilibrium, the system loses resilience.

Figure S3. Similar Network Dynamics Hide Different Node Dynamics. It shows different dynamic response at node level. While the mean dynamic shows the network is resilient, node 1 and node 2 have different dynamics. Node 1 only has one healthy equilibrium, but node 2 has a healthy equilibrium and an unhealthy equilibrium. Node 1 recover to the healthy state while node 2 remain in an unhealthy state at last. Therefore, the same network-level resilience may hide different node-level resilience. Estimation of resilience on node-level and network-level are both important.

Figure S4. Method to quantify the uncertainty of multi-scale resilience of networked system. The left part shows steps to quantify uncertainty of network-level resilience and the right part shows steps to quantify uncertainty of node-level resilience.


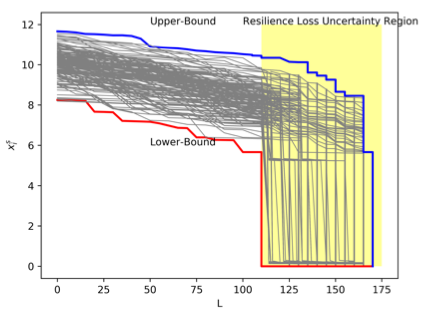


1. Resilience Bounds and Uncertainty Region with certain parameters

(b) Critical Resilience Value at Node Level with certainty parameters

Figure S5. Critical Resilience Value Identifies Vulnerable Nodes with Certain Parameters. (a) Resilience Bounds shows the Upper-Bound and Lower-Bound of equilibrium when links removed. In this figure, it explicitly predicts when the loss of resilience will happen. (b) Critical Resilience shows the relationship between average weight value of network and critical weight value. When $w_{i}^{\mathrm{in}}>w_{\mathrm{crit}}$, the node is resilient, otherwise it is not.

Figure S6. Approximate resilience of system by Polynomial Chaos Expansion. We truncate the series to arbitrary order $n$ from 2 to 5. (a) Approximate the minimum value of the system with uncertainty. It shows the result of approximating the minimum value of system by PCE with different truncate. (b) Probability of resilience. It shows the result of approximating the probability of resilience by PCE with different truncate. It is clear that there is a significant difference in results between $n=2$ and $n=3, 4, 5$ in (a) and (b).

Figure S7. Approximate the local minimum value $\tau^{i}$of node $i$ by aPC. These four subfigures (a) (b) (c) (d) respectively show the results of aPC truncate to different orders from 1 to 4. If node $\tau^{i}>0$, node $i$ is resilient. Otherwise, the node loses resilience.

(b) Probability of resilience when average weight of network is different

1. Probability of resilience when average weight of network is different


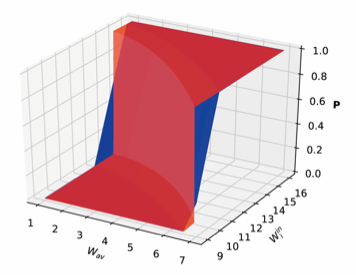


1. Relationship between critical weight and average weight with certain and uncertain parameters
2. Difference between certain and uncertain parameters when average weight of network changes

Figure S8. It shows the effect of uncertainty parameters on resilience of network and each node. (a) (b) show the probability of resilience at network-level and node-level in system with uncertainty. (c) (d) show the difference between resilience with certain parameters and uncertain parameters at network-level and node-level (Blue represents system with uncertain parameters and red represents system with certain parameters).

Figure S9. UK commuter network with urban job dynamics coupled by a competitive commuter mobility model. (a) Commuter Data from United Kingdom. Raw data consists of census data of home to work travel across 34,000 Lower Layer Super Output Areas (LSOA). This is then clustered to nearest major cities and the number of travellers inform the weight of the matrix. (b) Results on Uncertainty of Resilience. It simulated labour mobility dynamics with transport link removal causes simulated mobility dynamics to collapse.

**5.Supplement of Tables**

Table S1. Hermite polynomial

| Order | Polynomials |
| --- | --- |
| $H_{0}(x)$ | $1$ |
| $H_{1}(x)$ | $x$ |
| $H_{2}(x)$ | $x^{2}-1$ |
| $H_{3}(x)$ | $x^{3}-3x$ |
| $H_{4}(x)$ | $x^{4}-6x^{2}+3$ |
| $H_{5}(x)$ | $x^{5}-10x^{3}+15x$ |

Table S2. Methods to analyse resilience of system

| Method | Dimension of system | Interacting of components | Network-level resilience | Node-level resilience | Uncertainty of system | Limitations | Ref. |
| --- | --- | --- | --- | --- | --- | --- | --- |
| Performance-based methods with metrics | Low | No | Yes | No | No | Not suitable to high dimensional system with interacting components; Not consider network topology | 9, 10 |
| Network-based methods | High | Yes | Yes | No | No | Cannot estimate resilience of individual nodes; Not consider effects of uncertainty | 2, 11 |
| Sequential estimation method | High | Yes | No | Yes | No | Not consider effects of uncertainty on node-level and network-level resilience | 12 |
| Specific network- based method with PCE | High | Yes | Yes | No | Yes | Not consider effects of uncertainty on node-level resilience | 15 |

Table S3. Methods to estimate node-level resilience

| Method | Truncate order/Number of samples | Accuracy | Computational time (seconds) | Explainability |
| --- | --- | --- | --- | --- |
| Specific sequential estimation method with aPC | 1 | 0.5233 | 4.58 | Yes |
|  | 2 | 0.8224 | 4.89 |  |
|  | 3 | 0.9495 | 5.84 |  |
|  | 4 | 0.9502 | 6.11 |  |
| Specific sequential estimation method with Monte Carlo | 50 | 0.7549 | 7.9 | NO |
|  | 100 | 0.9299 | 10.9 |  |
|  | 400 | 0.9834 | 27.2 |  |
|  | 1000 | 0.991 | 68.79 |  |

Table S4. The number $Z$ of terms in polynomial expansion with different expansion order and variable number

| $l$ | $r$ | $Z$ | $l$ | $r$ | $Z$ |
| --- | --- | --- | --- | --- | --- |
| 2 | 2 | 6 | 3 | 2 | 10 |
|  | 3 | 10 |  | 3 | 20 |
|  | 4 | 15 |  | 4 | 35 |
|  | 5 | 21 |  | 5 | 56 |
| 4 | 2 | 15 | 5 | 2 | 21 |
|  | 3 | 35 |  | 3 | 56 |
|  | 4 | 70 |  | 4 | 126 |
|  | 5 | 126 |  | 5 | 252 |

**References**
1. Wei, Z. et al. Optimal sampling of water distribution network dynamics using graph fourier transform. IEEE Trans. Netw. Sci. Eng. 7, 1570–1582 (2020).
2. Gao, J., Barzel, B. & Barabási, A.-L. Universal resilience patterns in complex networks. Nature 530, 307–312 (2016).
3. Zhao, Y., Huepe, C. & Romanczuk, P. Contagion dynamics in self-organized systems of self-propelled agents. Sci. reports 12, 1–11 (2022).
4. Cohen, R., Erez, K., Ben-Avraham, D. & Havlin, S. Resilience of the internet to random breakdowns. Phys. Rev. Lett. 85, 4626 (2000).
5. Sole, R. V. & Montoya, M. Complexity and fragility in ecological networks. Proc. Royal Soc. B 268, 2039–2045 (2001).
6. Arghandeh, R., Von Meier, A., Mehrmanesh, L. & Mili, L. On the definition of cyber-physical resilience in power systems. Renew. Sust. Energ. Rev. 58, 1060–1069 (2016).
7. Li, Z., Zhao, H., Liu, J., Zhang, J. & Shao, Z. Evaluation and promotion strategy of resilience of urban water supply system under flood and drought disasters. Sci. reports 12, 1–21 (2022).
8. Kaiser-Bunbury, C. N. et al. Ecosystem restoration strengthens pollination network resilience and function. Nature 542, 223–227 (2017).
9. Hosseini, S., Barker, K. & Ramirez-Marquez, J. E. A review of definitions and measures of system resilience. Reliab. Eng. & Syst. Saf. 145, 47–61 (2016).
10. Cheng, Y., Elsayed, E. A. & Huang, Z. Systems resilience assessments: a review, framework and metrics. Int. J. Prod. Res. 1–28 (2021).
11. Zhang, Y., Shao, C., He, S. & Gao, J. Resilience centrality in complex networks. Phys. Rev. E 101, 022304 (2020).
12. Moutsinas, G. & Guo, W. Node-level resilience loss in dynamic complex networks. Sci. Rep. 10, 1–12 (2020).
13. Pulch, R. & ohters. Uncertainty quantification: introduction and implementations. In Nanoelectronic Coupled Problems Solutions, 197–221 (Springer, 2019).
14. Prince, Z. M. & Ragusa, J. C. Parametric uncertainty quantification using proper generalized decomposition applied to neutron diffusion. Int. J. Numer. Methods Eng. 119, 899–921 (2019).
15. Moutsinas, G., Zou, M. & Guo, W. Uncertainty of resilience in complex networks with nonlinear dynamics. IEEE Intell. Syst. 1–9 (2020).
16. Fishman, G. Monte Carlo: concepts, algorithms, and applications (Springer Science & Business Media, 2013).
17. Zhao, C. et al. Quantifying uncertainties of cloud microphysical property retrievals with a perturbation method. J. Geophys. Res. Atmos. 119, 5375–5385 (2014).
18. Zhang, D. Stochastic methods for flow in porous media: coping with uncertainties (Elsevier, 2001).
19. Wiener, N. The homogeneous chaos. Am. J. Math. 60, 897–936 (1938).
20. Shen, D. et al. Polynomial chaos expansion for parametric problems in engineering systems: A review. IEEE Syst J. 14, 4500–4514 (2020).
21. Wang, L., Chen, Z. & Yang, G. A polynomial chaos expansion approach for nonlinear dynamic systems with interval uncertainty. Nonlinear Dyn. 101, 1–20 (2020).
22. Abbaszadeh, M. et al. Uncertainty quantification in molecular signals using polynomial chaos expansion. IEEE Trans. Mol. Biol. Multi-Scale Commun. 4, 248–256 (2018).
23. Wackernagel, H. Multivariate geostatistics: an introduction with applications (Springer Science & Business Media, 2013).
24. Xiu, D. & Karniadakis, G. E. The wiener–askey polynomial chaos for stochastic differential equations. SIAM J. Sci.
Comput. 24, 619–644 (2002).
25. Xiu, D. & Karniadakis. Modeling uncertainty in flow simulations via generalized polynomial chaos. J. Comput. Phys. 187, 137–167 (2003).
26. Wan, X. & Karniadakis, G. E. Multi-element generalized polynomial chaos for arbitrary probability measures. SIAM J. Sci. Comput. 28, 901–928 (2006).
27. Oladyshkin, S. & Nowak, W. Data-driven uncertainty quantification using the arbitrary polynomial chaos expansion. Reliab. Eng. Syst. Saf. 106, 179–190 (2012).

28. May, R. M. Thresholds and breakpoints in ecosystems with a multiplicity of stable states. Nature 269, 471–477 (1977).
29. Lyapunov, A. M. The general problem of the stability of motion. Int. J. Control. 55, 531–534 (1992).
30. Oladyshkin, S., Class, H., Helmig, R. & Nowak, W. A concept for data-driven uncertainty quantification and its application to carbon dioxide storage in geological formations. Adv. Water Resour. 34, 1508–1518 (2011).
31. Lundqvist, M., Compte, A. & Lansner, A. Bistable, irregular firing and population oscillations in a modular attractor memory network. PLoS Comput. Biol. 6 (2010).
32. Todman, L. et al. Evidence for functional state transitions in intensively-managed soil ecosystems. Sci. Rep. 8, 1–10 (2018).
33. Marsh, R. et al. Bistability of the thermohaline circulation identified through comprehensive 2-parameter sweeps of an efficient climate model. Clim. Dyn. 23, 761–777 (2004).
34. Aquino, G., Guo, W. & Wilson, A. Nonlinear dynamic models of conflict via multiplexed interaction networks. Prepr. arXiv 1909.12457 (2019).
35. Ron, J. et al. Bi-stability in cooperative transport by ants in the presence of obstacles. PLoS Comput. Biol. 14, e1006068 (2018).
36. Wilson, A. Boltzmann, lotka and volterra and spatial structural evolution: an integrated methodology for some dynamical systems. J. R. Soc. Interface 5, 865–871 (2008).
37. Holland, J. N., DeAngelis, D. L. & Bronstein, J. L. Population dynamics and mutualism: functional responses of benefits and costs. Am. Nat. 159, 231–244 (2002).
38. Allee, W. C. et al. Principles of animal ecology. Tech. Rep., Saunders Company Philadelphia, Pennsylvania, USA (1949).
39. Pagani, A. et al. Resilience or robustness: identifying topological vulnerabilities in rail networks. Royal Soc. Open Sci. 6 (2019).
